# Enhancing the Performance of Dye-Sensitized Solar Cells with a Three-Layer Photoanode

**DOI:** 10.3390/ma19071286

**Published:** 2026-03-24

**Authors:** Zhou Li, Lihua Bai, Yuan Zhang, Zhangyang Zhou, Teng Zhang

**Affiliations:** College of Intelligent Systems Science and Engineering, Hubei Minzu University, Enshi 445000, China

**Keywords:** dye-sensitized solar cells, photoanode, light harvesting

## Abstract

Dye-sensitized solar cells (DSCs) have garnered significant attention due to their high power conversion efficiency and low production cost-effectiveness. In this study, we developed a hierarchically structured three-layer TiO_2_ photoanode via hydrothermal synthesis to significantly enhance DSC performance. The optimized device achieved a short-circuit current density of 16.92 mA/cm^2^ and a photoelectric conversion efficiency of 8.34%, representing improvements of 15.67% and 20.5%, respectively, compared to traditional DSCs with a single-layer TiO_2_ photoanode in our study. The significance lies in the rational design principle rather than absolute efficiency. This performance enhancement stems from the complementary functions of each architectural layer: (1) a bottom layer of TiO_2_ nanocrystals providing high surface area for dye adsorption, (2) an intermediate layer of vertically aligned TiO_2_ nanorods enabling efficient electron transport, and (3) a top layer of TiO_2_ microspheres simultaneously boosting dye loading and light harvesting through enhanced light scattering. Our findings demonstrate that rational design of multi-layered photoanode architectures can effectively address the competing demands of surface area, charge transport, and light management in high-performance DSCs.

## 1. Introduction

Since the Industrial Revolution, the escalating demand for energy has led to a continuous increase in the consumption of fossil fuels, fundamentally altering the energy structure. Clean energy sources such as solar, nuclear, and wind energy now hold significant positions in the new energy structure. While perovskite and silicon solar cells have achieved remarkable efficiencies, dye-sensitized solar cells (DSCs) remain attractive due to their low cost, flexibility, and aesthetic potential [1,2]. DSCs, reported in 1991, have garnered widespread attention from scholars in related fields [1,2,3,4,5]. DSC devices typically feature a “sandwich structure” [6,7,8], composed of a photoanode, electrolyte, and counter electrode. The photoanode is the core component of DSC devices, with the cell performance being closely related to the specific surface area of the photoanode film, the strength of light scattering, and the speed of carrier transport [9,10,11]. An ideal photoanode should possess the following characteristics: (1) a large specific surface area to ensure a higher dye loading capacity; (2) strong light scattering effects to enhance light-harvesting performance; (3) rapid electron transport rates to ensure high electron collection efficiency [12,13]. However, it is challenging for a single type of photoanode to simultaneously possess all three of these characteristics. Traditional DSC devices feature photoanodes composed of TiO_2_ nanocrystalline particles, approximately 15 to 20 nm in size. While these offer a relatively high specific surface area to ensure dye adsorption, the small size of the TiO_2_ nanocrystalline particles results in a relatively transparent photoanode film with weak light scattering, leading to a low light capture efficiency for the incident light by the photoanode film layer. Moreover, the disordered network formed by the TiO_2_ nanocrystalline particles induces electron transfer through a “random walk” mechanism [14], reducing electron mobility and increasing the probability of carrier recombination, thereby limiting the photoelectric conversion efficiency of DSC devices.

In recent years, scholars have prepared one-dimensional nanostructured titanium dioxide (such as nanorods, nanowires, and nanotubes [15,16]) to improve carrier transport efficiency and enhance light scattering effects. Unlike the disordered network formed by TiO_2_ nanocrystalline particle films, one-dimensional ordered nanoarrays provide a direct pathway for electron transport, accelerating carrier transfer and effectively reducing carrier recombination. Therefore, one-dimensional nano materials typically exhibit higher carrier diffusion coefficients and stronger light scattering compared to disordered nanocrystalline particles. Despite these advantages, the smaller specific surface area of one-dimensional nano materials limits dye loading. It has been reported that the introduction of larger particle nanocrystals can also enhance the scattering effect [17,18,19], but the addition of large particles reduces the effective area and dye loading capacity. In summary, if the advantages of different TiO_2_ configurations can be fully utilized to form a composite photoanode with multiple functional layers, leveraging the complementary strengths of each functional layer could enhance the performance of the photoanode and DSC devices. Beyond 1D structures, other challenging TiO_2_ architectures, such as engineered multi-scale or doped nanostructures, have also shown promise in optimizing light management and charge dynamics, highlighting the continuous innovation in photoanode design [2].

Based on the above considerations, this paper develops a novel three-layer photoanode structure: the bottom layer consists of zero-dimensional TiO_2_ nanocrystals, the middle layer is a one-dimensional array of TiO_2_ nanorods, and the top layer is three-dimensional TiO_2_ microspheres. The high specific surface area of the bottom layer TiO_2_ nanocrystals ensures high dye adsorption, the middle layer of TiO_2_ nanorod arrays serves as a rapid electron pathway to improve carrier transport efficiency, and the top layer of three-dimensional TiO_2_ microsphere structures enhances light scattering to increase light absorption. To compare with the novel three-layer photoanode, single-layer TiO_2_ nanocrystal and three types of double-layer film structures (nanocrystal-nanorod, nanocrystal-microsphere, nanorod-microsphere) photoanode films were prepared, and the performance of the above single-layer, double-layer, and three-layer structured photoanode films, as well as the photoelectric characteristics of their DSC devices, were systematically studied. In this work, we propose and demonstrate a rational, solution-processable design rule for multi-layered photoanodes. By sequentially stacking distinct TiO_2_ morphologies, nanoparticles, nanorods, and microspheres, we aim to synergistically optimize surface area, charge transport, and light scattering within a single electrode. This strategy provides a clear architectural blueprint that can be extended to other photovoltaic and photoelectrochemical devices.

## 2. Experimental Sections

### 2.1. Film Growth and Device Fabrication

The glacial acetic acid (2.1 g, Sigma-Aldrich (St. Louis, MO, USA), ≥99.8%) was added to 10 mL of titanium isopropoxide (Aladdin (Singapore), 97%) and stirred for 30 min. Subsequently, 50 mL of deionized water was added to the solution with vigorous stirring. 0.68 mL of nitric acid was then added to the mixture, followed by hydrolysis for 4 h at 80 °C in a water bath, resulting in a white transparent solution. This solution was diluted to approximately 70 mL and placed in a 100 mL autoclave, which was then heated at 220 °C for a period of time. The resulting stratified liquid was removed, and 0.4 mL of nitric acid was added with vigorous stirring for 1 h. A large amount of anhydrous ethanol was then added, followed by high-speed centrifugation to isolate the titanium dioxide solid, which was subsequently dried. A certain amount of the dried powder was added to a ball mill jar, along with ethyl cellulose and terpineol, and milled for ten hours. The resulting solution was then ultrasonicated for 30 min. This process was repeated three times, and the milled slurry was then rotary evaporated to yield the TiO_2_ nanocrystal slurry. The approximate thicknesses of each layer, controlled by paste volume and confirmed by cross-sectional SEM, were: TiO_2_ nanoparticle layer: ~6 µm, nanorod layer: ~4 µm, and microsphere layer: ~4 µm, yielding a total photoanode thickness of ~14 µm.

Hydrochloric acid (30 mL) was mixed with deionized water (30 mL) and stirred for 5 min. 1 mL of titanium tetrabutoxide was added and stirred for an additional 5 min. The mixture was then transferred to a hydrothermal reactor, and a titanium dioxide membrane that had been coated with the first layer of particles was suspended face-down in the reactor. The hydrothermal reaction was carried out at 150 °C for 3 h. The resulting product was rinsed with deionized water and dried for later use.

The titanium tetrabutoxide (20 mL) was added dropwise to glacial acetic acid (60 mL) and sonicated for 15 min. The mixture was then transferred to a hydrothermal reactor and reacted at 160 °C for 8 h. After cooling, the solution was removed, washed several times with anhydrous ethanol, and dried to yield TiO_2_ microsphere powder. 1 g of the titanium dioxide microsphere powder was mixed with 5 mL of terpineol, 0.05 g of lauric acid, and 0.2 g of ethyl cellulose until uniform, resulting in the TiO_2_ microsphere slurry.

The TiO_2_ nanocrystal slurry was coated onto conductive glass (FTO) using a doctor-blade method, dried, and then annealed at 500 °C for half an hour. A layer of titanium dioxide nanorod arrays was grown on this film, which was then dried and annealed. The titanium dioxide microsphere slurry was then coated onto the second layer using the same method, dried, and annealed to obtain the three-layer photoanode film, labeled as TRS. The same method was used to coat the titanium dioxide nanoparticle slurry onto FTO to prepare a single-layer TiO_2_ nanocrystal film, labeled as T. On the T film, one-dimensional titanium dioxide nanorod array films and titanium dioxide microsphere slurry films were prepared separately to obtain the double-layer structures, labeled as TR and TS, respectively. A one-dimensional titanium dioxide nanorod array was directly prepared on FTO, followed by the coating of a layer of titanium dioxide microsphere slurry to obtain the double-layer structure, labeled as RS.

### 2.2. Characterizations and Measurements

The morphology of one-dimensional titanium dioxide nanorods, titanium dioxide microspheres, and the three-layer titanium dioxide photoanode films was characterized using scanning electron microscopy (SEM, Hitachi SU-8000 (HITACHI, Tokyo, Japan), operated at 5–10 kV accelerating voltage and ~8 mm working distance). Optical spectra were measured using a UV-Vis spectrophotometer (Shimadzu UV-2600, Shimadzu, Kyoto, Japan). The scattering and transmission spectra of the photoanode films before sensitization, as well as the absorption spectra of the films after sensitization, were measured using a UV-Vis spectrophotometer. All sensitized photoanode films were sequentially immersed in solutions of NaOH at specific concentrations to desorb the dye, and the absorbance of these solutions was measured using a UV-Vis spectrophotometer to calculate the amount of dye adsorbed. The relative dye loading was quantified by integrating the area under the characteristic absorption peak of the desorbed dye solution (around 500 nm) and is reported as an integrated absorbance value (a.u.). Under irradiation from a simulated light source with an intensity of 100 mW/cm^2^ (AM 1.5), an electrochemical workstation was used to collect the *J*-*V* performance curves (using a consistent scan direction to minimize hysteresis effects) and electrochemical impedance spectra (EIS) of the cells, with the illuminated area of the cells controlled to 0.25 cm^2^ using a light shield.

## 3. Results and Discussion

**Figure 1:**
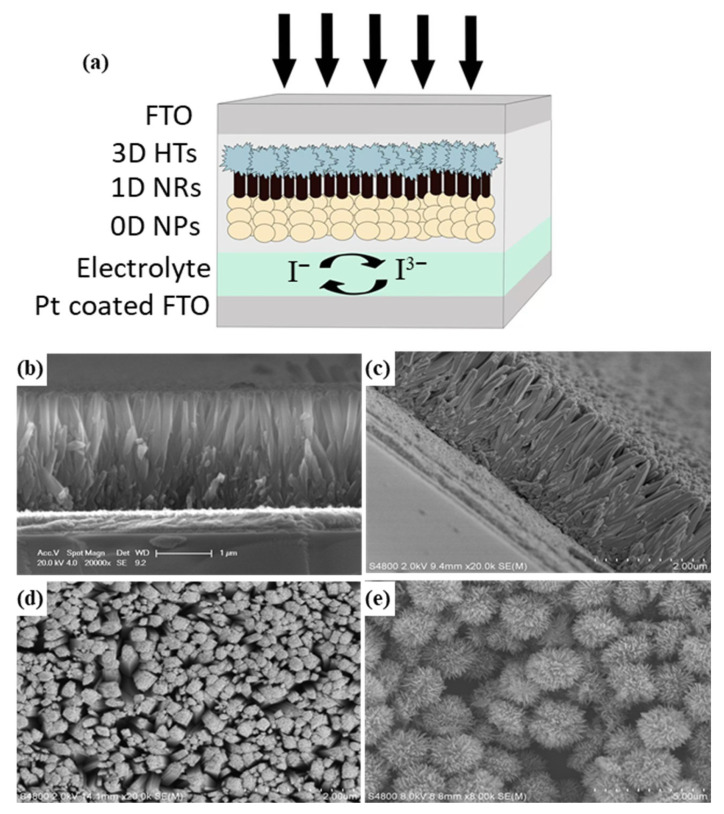
(**a**) schematic diagram of the device structure, SEM images of oriented TiO_2_ nanorod film (**b**) cross-sectional view, (**c**) tilted cross-sectional views, (**d**) top view. (**e**) SEM image of TiO_2_ flower spheres.

Figure 1a presents a schematic diagram of the device structure, which incorporates a three-layer TiO_2_ thin film as the photoanode. The bottom layer consists of dense small TiO_2_ nanoparticles, which enlarge the contact area and contact sites between the TiO_2_ film and the FTO substrate, effectively blocking the direct contact between the electrolyte I^3−^ and FTO and thereby suppressing charge recombination at the FTO/TiO_2_ interface. The middle layer is composed of one-dimensional TiO_2_ nanorods arranged in a close and orderly manner, serving as a bridge between the upper and lower layers to accelerate electron transport; moreover, rutile-phase TiO_2_ nanorods exhibit a higher refractive index than their anatase-phase counterparts, which can enhance light scattering to a certain extent. The top layer comprises TiO_2_ flower-like microspheres, which are interlocked with one another through their petal-like protrusions, increasing the surface roughness and connectivity, and this hierarchical structure not only enhances light absorption and scattering but also facilitates electron transport. Figure 1b–d displays the scanning electron microscopy (SEM) images of the titanium dioxide nanorod array films, revealing densely packed and vertically aligned nanorods with an average diameter of ~50 nm and length of ~1 µm. Figure 1b is a cross-sectional view, Figure 1c is a tilted cross-sectional view, and Figure 1d is a top view. Images from different positions show that the nanorods are closely packed and uniformly oriented perpendicularly. Figure 1e presents the SEM image of the titanium dioxide microspheres. The image clearly shows that the titanium dioxide microspheres are highly uniform in size and well-dispersed, with a flower-like morphology. The formation of this specific flower-like morphology is primarily dictated by the hydrothermal conditions which favor oriented attachment and self-assembly.

**Figure 2:**
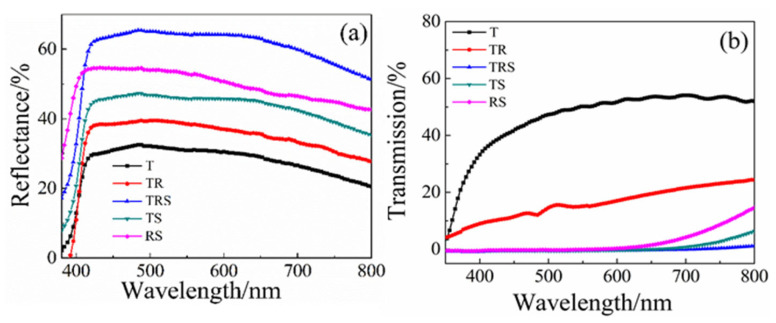
(**a**) Diffuse reflectance spectra of the photoanode films. (**b**) Transmittance spectra measured through the complete assembled DSC devices.

Figure 2a shows the scattering spectra of different structured photoanode films. It can be observed from Figure 2a that the three-layer photoanode (TRS) and the two-layer photoanodes (TR, TS, RS) exhibit higher scattering properties compared to the single-layer (T) photoanode. This is associated with the introduction of larger particle TiO_2_ microspheres and the anatase phase one-dimensional titanium dioxide nanorods. The three-layer photoanode structure (TRS) demonstrates superior scattering performance compared to the other types of photoanode films. This is primarily attributed to the second layer of nanorods and the third layer of microspheres, which increase the path of light scattering within the film, confining more light within the photoanode and significantly reducing light transmission. Figure 2b presents the transmission spectra of different films. The results indicate that the single-layer film composed of small titanium dioxide particles has the highest light transmittance, while the three-layer structured film is almost opaque, illustrating the strong light scattering and light-harvesting effects of the top two layers. The optical band gap of the primary TiO_2_ materials was estimated using the Tauc plot method from diffuse reflectance data, yielding values of ~3.2 eV, consistent with the anatase phase.

**Figure 3:**
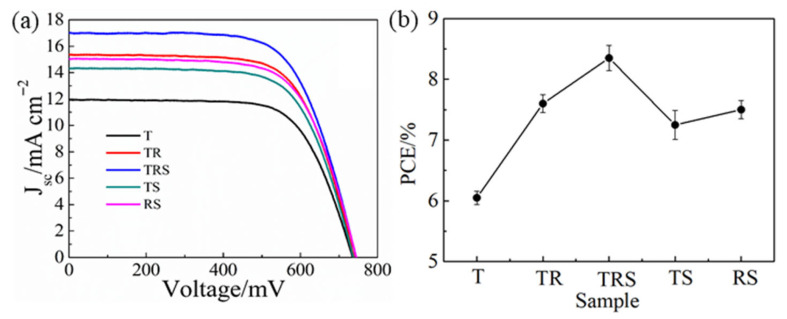
The J-V curves (**a**) and the dependence of the PCE (**b**) of DSC-based different photoanodes.

The *J*-*V* characteristic curves of the DSC devices based on the five types of photoanode structures are shown in Figure 3, with the corresponding performance metrics and parameters detailed in Table 1. Among these, the DSC device with a single-layer photoanode composed of TiO_2_ nanoparticles exhibited the lowest conversion efficiency (6.04%) and photocurrent (11.94 mA∙cm^−2^). This is attributed to the thick, disordered film of TiO_2_ nanoparticles, which hinders electron transport through the film, leading to electron recombination before reaching the FTO glass, thereby reducing the short-circuit current density. Devices with double-layer photoanode structures showed improved performance compared to the single-layer structure. The DSCs with a three-layer photoanode structure achieved the highest conversion efficiency and photocurrent, at 8.34% and 16.92 mA∙cm^−2^, respectively. This enhancement is primarily due to the synergistic effects of each layer in the TRS structure: the bottom layer of small nanoparticles ensures adequate dye adsorption and better interface contact with the FTO; the middle layer of nanorods enhances electron transport; the top layer of microspheres, with a specific surface area comparable to that of the bottom layer, not only ensures dye adsorption but also enhances light scattering.

**Figure 4:**
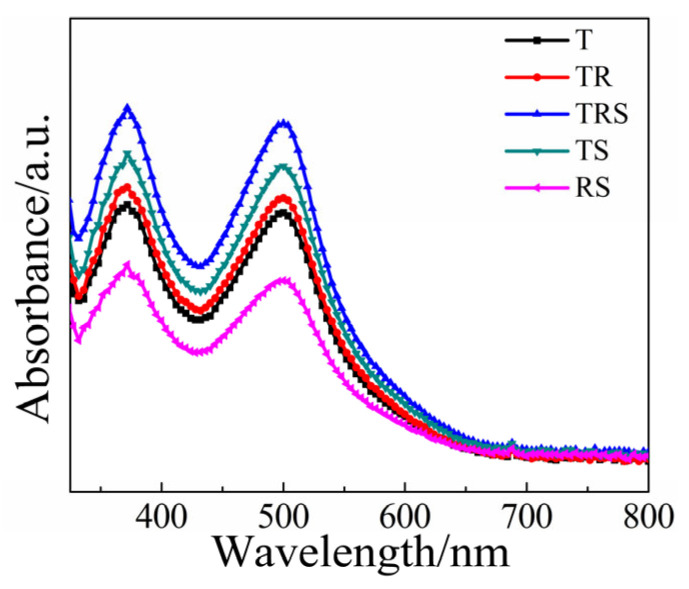
Absorption spectra after desorbed from different photoanodes.

To investigate the reasons behind the variations in performance parameters among the five types of batteries, further dye adsorption tests were conducted on all five batteries. Figure 4 presents the UV-Vis absorption spectra of the desorbed dye from the sensitized photoanodes after soaking in NaOH solution, from which the dye adsorption amounts of each photoanode were calculated and the results are listed in Table 1. It is evident that only the single-layer film T, composed of small TiO_2_ particles, possesses the highest dye loading capacity; the three-layer photoanode TRS has a slightly lower dye loading capacity compared to the single-layer T (2.54 ± 0.15 vs. 2.83 ± 0.15). This is primarily attributed to the lower specific surface area of the introduced one-dimensional nanorod array. Although the single-layer structure composed of TiO_2_ nanoparticles has the highest dye adsorption and light-harvesting efficiency, the disordered network of small particles affects electron transport within the titanium dioxide, leading to a significant recombination of electrons with the electrolyte before they reach the FTO, reducing electron collection efficiency and thus resulting in the lowest photoelectric conversion efficiency. The three-layer photoanode structure effectively overcomes this drawback; the bottom layer of small particles and the top layer of microspheres ensure adequate dye adsorption, the middle layer of nanorods provides a direct pathway for electron transport, and the large microspheres on the top layer enhance light scattering to ensure efficient light capture. Consequently, this three-layer photoanode structure can improve battery performance from multiple aspects simultaneously.

**Figure 5:**
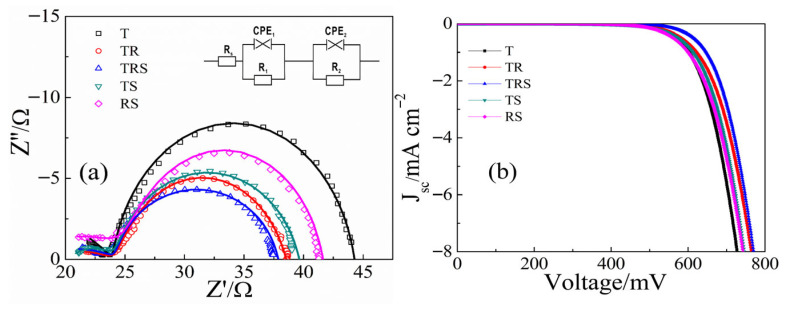
(**a**) EIS spectra and (**b**) Dark current–voltage characteristics of the DSCs based on different photoanodes.

Electrochemical impedance spectroscopy (EIS) was employed to analyze the electron transport properties of DSC devices based on different structured photoanodes [20,21], to further investigate the mechanisms by which different photoanode structures enhance cell performance. The Nyquist plots obtained under illumination for each device are shown in Figure 5a. These curves were fitted using EIS Spectrum Analyser (version 1.0) with the equivalent circuit diagram (inset in Figure 5a), where symbols represent experimental data and lines represent fitted data. The internal resistance values obtained for the devices are listed in Table 1. In the EIS plots, the transfer resistance R_1_, reflecting the Pt counter electrode/electrolyte interface, is not considered as the same Pt counter electrode was used throughout the experiment. Rs represents the contact resistance between the FTO and the titanium dioxide film. The Rs values for the T, TR, TRS, and TS films are similar because their bottom layers consist of dense TiO_2_ nanoparticles in contact with FTO. In contrast, the RS film, with its bottom layer of one-dimensional nanorods, exhibits poor contact with FTO, resulting in the highest R_s_ value among the films. This further validates the advantage of designing the bottom layer of the three-layer photoanode as dense small particles. R_2_ reflects the charge transfer resistance at the TiO_2_/dye/electrolyte interface. The TRS device exhibits the smallest R_2_ value, indicating the best electron transport characteristics. This is not only due to the good contact between the dense TiO_2_ nanolayer at the bottom and the FTO but also because the middle layer of TiO_2_ nanorods acts as a “bridge” facilitating rapid electron transport, resulting in the smallest R_2_. This well-designed three-layer photoanode structure can improve cell performance in multiple aspects simultaneously.

To investigate the backflow of photogenerated carriers and their recombination with I^3−^, dark current tests were conducted on DSCs using different photoanodes. As shown in Figure 5b, the DSCs with the three-layer photoanode structure exhibit the smallest dark current, indicating the least carrier backflow and recombination loss, which results in a larger short-circuit photocurrent. This result is consistent with the *J*-*V* curve variations. The improved carrier recombination loss situation is mainly attributed to the good contact at the bottom layer and the rapid carrier migration pathway provided by the middle nanorod layer. Comparing the performance of our best TRS device with other representative multi-layered or hierarchical TiO_2_ photoanodes from recent literature [22,23,24,25,26,27,28], we highlight the competitive efficiency achieved through our rational structural design.

## 4. Conclusions

The novel three-layer photoanode structure comprises TiO_2_ nanoparticles as the bottom layer, one-dimensional titanium dioxide nanorods as the middle layer, and large TiO_2_ microspheres as the top layer. This photoanode has been successfully fabricated and applied in dye-sensitized solar cells (DSCs). The results demonstrate that the DSCs with the three-layer structure exhibit superior photoelectric performance compared to other single-layer and double-layer structured DSCs, with the advantages of efficient charge collection, light harvesting, and high dye loading. A maximum conversion efficiency of 8.34% was achieved for the TRS device, representing a significant (38%) improvement over the single-layer baseline in our study. More importantly, this work establishes a clear design principle where the rational integration of complementary morphologies (NPs for surface area, NRs for transport, MSs for scattering) leads to balanced and enhanced performance. While absolute efficiency can be further boosted by combining this architecture with advanced dyes and electrolytes, our findings provide a reliable and scalable architectural strategy for next-generation DSC photoanodes. Preliminary stability testing indicated that the champion TRS device retained approximately 92% of its initial PCE after one week of storage in the dark under ambient conditions, suggesting promising robustness for the architecture. This enhancement is primarily attributed to the three-layer photoanode structure, which consists of transparent TiO_2_ nanoparticles, a TiO_2_ nanorod array that effectively transports photogenerated electrons and reduces recombination, and TiO_2_ microspheres that efficiently scatter incident light. The bottom layer of nanoparticles has a large specific surface area and forms good contact with the FTO, while the top layer of large microspheres provides excellent light scattering. This composite multifunctional structure significantly improves the overall performance of the cell. Therefore, through rational structural design, the advantages of each layer can be fully utilized to effectively enhance the photoelectric performance of DSC devices.

## Figures and Tables

**Table 1:** Performance parameters of DSSC based on different photoanodes.

DSCs	*J*_sc_/ (mA∙cm^–2^)	*V*_oc_/ (mV)	*FF*	*η*/ %	*R*_s_/ (Ω)	*R*_2_/ (Ω)	Absorbance/ (×10^−7^ mol∙cm^−2^)
T	11.94	735	0.68	6.04	18.22	20.32	2.83 ± 0.06
TR	15.35	738	0.68	7.68	18.00	13.4	2.47 ± 0.03
TRS	16.92	740	0.66	8.34	16.99	11.29	2.54 ± 0.05
TS	14.31	736	0.68	7.15	17.80	14.00	2.69 ± 0.04
RS	15.01	744	0.67	7.52	23.05	16.63	2.12 ± 0.03

## Data Availability

The original contributions presented in this study are included in the article. Further inquiries can be directed to the corresponding authors.
